# A Rare Case of Ureteral Injury: Fall from Standing

**DOI:** 10.51894/001c.144469

**Published:** 2025-10-01

**Authors:** Alexander Haberman

**Affiliations:** 1 Department of Urology University of Michigan Health Sparrow

## 85

Ureteral injury is a rare form of genitourinary trauma, only occurring in about 1% of GU injuries. Most are associated with penetrating trauma with gunshot wounds accounting for 55% of injury with usually concomitant bowel and vascular injuries. Blunt injury is less common and typically associated with high velocity deceleration such as a MVC with concomitant pelvic and spine fractures. Iatrogenic injury also occurs during surgical procedures, it is rarely reported for a low velocity injury to cause injury. A pubmed search was conducted for ureteral trauma with low velocity injury exceedingly rare. An 86yo male presented to the ER due to left sided chest pain after a fall from standing. Imaging demonstrated multiple rib fractures as well as a 4.5cm intraluminal bladder mass, no suspicious renal findings or other fractures. After placement of a large bore catheter with subsequent irrigation of clot, subsequent CT cystogram and delayed abdominal imaging demonstrated no bladder lesions with contrast extravasation along the ureter concerning for ureteropelvic junction injury. Patient was emergently taken for ureteral stent placement. Cystoscopy demonstrated formed blood clot within the bladder with no mass. Subsequent retrograde pyelogram demonstrated extravasation of contrast along the left UPJ and renal pelvis confirming UPJ injury and a stent was successfully placed. Fortunately an injury like this can be managed without major abdominal surgery but early detection is key to prevent future complications such as urinoma and persistent leak. This case serves as a precautionary tale to have a low threshold to suspect GU injury in the setting of gross hematuria regardless of mechanism of trauma. Although rare, ureteral injury must be ruled out in this setting.

